# Verification with the utility of an established rapid assessment of brain safety for newly developed vaccines

**DOI:** 10.1186/s42826-019-0027-8

**Published:** 2019-11-29

**Authors:** Gwang-Ho Kim, Sun Shin Yi

**Affiliations:** Department of Biomedical Laboratory Science, Soonchunhyang University, Asan, 31538 Republic of Korea

**Keywords:** Brain, Infectious disease, Public health, Rapid assessment, Vaccine safety

## Abstract

In the twenty-first century, high contagious infectious diseases such as SARS (Severe Acute Respiratory Syndrome), MERS (Middle East Respiratory Syndrome), FMD (Foot-and-Mouth Disease) and AI (Avian Influenza) have become very prevalent, causing treat harm to humans and animals in aspect of public health, and economical issues. The critical problem is that newly-reported infectious diseases that humans firstly experience are expected to continue to emerge, and these diseases will be spreading out rapidly. Therefore, rapid and safe supplies of effective vaccines are most pivotal to prevent the rapid prevalent of new infection, but international standards or assessing protocol the safety of urgent vaccines are not established well. In our previous study, since we established a module to assess the brain safety of urgent vaccines, therefore, it is necessary to verify that this established module for assessing brain safety could work effectively in commercially available two vaccines (one killed- and on live-vaccines). We compared the results of Evans blue (EB) assay and qPCR analysis by injection of two kinds of vaccines, PBS and Lipopolysaccharide (LPS) under the condition of the module previously reported. We confirmed that the brain safety test module for urgent vaccine we established is very reproducible. Therefore, it is believed that this vaccine safety testing method can be used to validate brain safety when prompt supply of a newly developed vaccines is needed.

## Introduction

Recently, a variety of infectious diseases with high rates of spread and mortality have been frequent. In addition, it has been reported that high contagious infectious diseases are spreading through various routes and carriers for recent two decades. It is important to prevent from spreading out a number of infectious diseases earlier in the outbreak of the causing agents because of our rapid transportation population density. Therefore, prompt supplying of vaccines should be secured within a limit time, even at unexpected times and places. The speed of vaccine development is also a very critical issue. Thus, safety of the vaccines also should be assured. Nevertheless, it is difficult to obtain sufficient time to perform a complete safety evaluation for a rapid supply of urgent vaccines. It is very unfortunate that we have already mentioned in the previous report that there is not global standard for the safety assessment of these newly develop urgent vaccines.

In our previous study, we established of minimal positive control conditions to ensure brain safety using experimental mice models during development of urgent vaccines before introducing to markets. We conducted several experiments to evaluate the safety of vaccines, and presented the ideal combination of the experiments: susceptible mice types according to the effective concentration of lipopolysaccharide (LPS) destroying Blood-brain barrier (BBB), the appropriate LPS exposure time destroying BBB, origin type of LPS (*Salmonella enterica*), and injection route of LPS. In this study, we tried to evaluate whether the previously established assessment method could be available for evaluating brain safety rapidly with two commercial vaccines easily accessible vaccines (a killed- and a live-vaccine). We confirmed in the present study that the pre-established “vaccine safety module for the brain” was highly reproducible in the evaluation of the safety results by the commercially available vaccines.

In this study, we described that this module can be useful in applying a commercially available vaccine to a rapid and effective positive-control for brain safety assessment of an urgent vaccine. This study will be of great help to ensure that vaccines should be urgently and promptly distributed to public with assured minimal safety of brain at an unexpected outbreak of certain infectious diseases in human and veterinary fields.

## Materials and methods

Most of experiments were performed based on our previous study. Among the experiments, we primarily chose some combinations that we’d suggested in the previous reports by Baek et al., [1].

In brief, evidences for the BBB damage by commercially available vaccines (killed porcine genital respiratory syndrome vaccine vaccine and live canine parvovirus vaccine) were based on Evans blue EB permeability into brain parenchyma and mRNA expression of tight junctions (ZO-1 and Occludin) in the brain. Experimental method for this is described in detail below.

### Evans blue (EB) assay for brain permeability

Seven-weeks old ICR mice and LPS sources *Salmonella enterica* were used. LPS solutions concentration was 1.0 mg/4 ml/kg, and i.p injection was performed. The LPS from *S.enterica* serotype *enteritidis* (L6011; Sigma, USA) was dissolved in phosphate buffered saline (PBS; pH 7.4). Two types of commercially available vaccines were used as experimental groups. One is a killed vaccine, a porcine genital respiratory syndrome vaccine (Suishot PRRS, Choongang vaccine, Korea). The other one is a live vaccine, a canine parvovirus vaccine (Zoetis Inc., USA). The concentration of the stock solution of these two vaccines is 0.5 × 10_7.0_ TCID_50_/ml. We set these concentrations to assess the effect of these vaccines on the BBB, depending on the concentration. Through the previous studies we confirmed that the effective BBB destroyed concentration of these vaccines was 2-fold concentration of the stock solutions. Therefore, the experiment was conducted under three conditions: 2-fold concentration of the stock solution, reference concentration of the stock solution and 10-fold diluted concentration of the stock solution. Including negative control and positive control, the groups were divided 8 groups according to vaccine types and concentrations: group 1, PBS/EB(*n* = 2); group2, LPS/EB(n = 2); group3, 2-fold concentration of the porcine vaccine/EB (P1, *n* = 5); group4, reference concentration of the porcine vaccine/EB (P2, *n* = 5); group5, 10-fold diluted concentration of the porcine vaccine/EB (P3, *n* = 5); group6, 2-fold concentration of the canine vaccine/EB (C1, *n* = 5); group7, reference concentration of the canine vaccine/EB (C2, *n* = 5); group8, 10-fold diluted concentration of the canine vaccine/EB (C3, n = 5). We performed the EB assay according to the method of Baek et al. [1], used in previous studies.

For treatment, PBS, LPS and the vaccines were intraperitoneally (i.p) injected into each mouse (1.0 mg/4 mL/kg body weight). At 1 h post-injection (PI) of PBS/LPS/Vaccines, EB dye (2% w/v in PBS, 4 mL/kg, E2129; Sigma) was i.p injected into each mouse. All mice were sacrificed after 3 h of EB exposure (4 h PI). After sacrifice, blood was collected and serum was separated by using centrifugation. The brain was promptly removed after the mouse had been perfused with heparinized PBS (0.1 mg/L). Serum and homogenized brain hemisphere of each mouse were dissolved in a 50% (w/v) trichloroacetic acid in PBS solution to eliminate proteins and then subjected to centrifugation at 5000×*g* for 20 min. Supernatant of each sample was diluted 1:3 with absolute ethanol.

A spectrophotometer (Infinite F200; Tecan, Switzerland) was used to determine the EB concentrations in the serum and brain hemisphere samples. The fluorescence intensity was measured at 620λ_ex_ and 680_em_, and the EB concentration of each sample was calculated according to a standard curve that had been ordered to determine the penetration rate for EB in brain to EB in blood was calculated.

### Brain tissue collection and preparation of RNA samples

Following the EB assay, the safety of the vaccine was assessed by mRNA expression of the molecules forming the tight junction of the brain. Groups and experimental conditions were used same as EB assay. The LPS solution was i.p. injected into all mice and sacrificed at the 4 h PI time. After sacrifice, the brains were removed from each mouse and were prepared for next steps. RNA was extracted by using Trizol reagent (Ambion; Life Technologies, USA) according to the manufacture’s instruction. For RNA extraction, the homogenized brain tissue was incubated in Trizol and chloroform reagent and then subjected to centrifugation. The aqueous phase was removed and incubated with 100% isopropanol. After centrifugation, the obtained RNA pellet was washed with 75% ice-cold ethanol and then dissolved in RNase-free water. The obtained RNA extract solution was stored at − 70 °C after undergoing 60 °C heat incubation.

### Quantification of mRNA expression of ZO-1 and occluding in the brain

Quantitative real-time polymerase chain reaction (qPCR) was performed with SYBR Green dye by using a Step One Plus Real-time PCR system (Life Technologies). For relative quantitation of gene expression, we used the comparative cycle threshold (Ct) method (2^-△△Ct^). Results were normalized to that of the housekeeping/control gene glyceraldehyde 3-phosphate dehydrogenase (GAPDH). The primer sequences for zonula occludens-1 (ZO-1) and occludin have been used in our previous studies to establish positive control. These primer sequences were obtained from the National Center for Biotechnology Information nucleotide database and are shown in Table 1.

**Table 1:** Primer Sequences of ZO-1, occludin, and GAPDH

Target mRNA	Sequences
ZO-1	Forward 5′ – ACA GGC CAT TAC GAG CCT CT − 3′
	Reverse 5′ – GGA GGC TGT GGT TTG GTA GC − 3’
Occludin	Forward 5′ – CAC ACA GGA CAT GCC TCC AC − 3’
	Reverse 5′ – GGC TGC CTG AAG TCA TCC AC − 3’
GAPDH	Forward 5′ – GAC GGC CGC ATC TTC TTG T-3’
	Reverse 5′ – CAC ACC GAC CTT CAC CAT TTT − 3’

Sequences of primers used for quantitative real-time PCR analysis. The sequence of the primers was used with reference to the previous description of Baek et al. [1]. ZO-1, zonula occludens-1; GAPDH, glyceraldehyde 3-phosphate dehydrogenase

### Data analysis

Data are presented as mean ± SE or mean ± SEM values for each experimental group. Differences between means were analyzed by using Student’s *t-*test for single comparisons. *P* values< 0.05 were considered statistically significant.

## Results

### EB assay for brain permeability

**Fig. 1:**
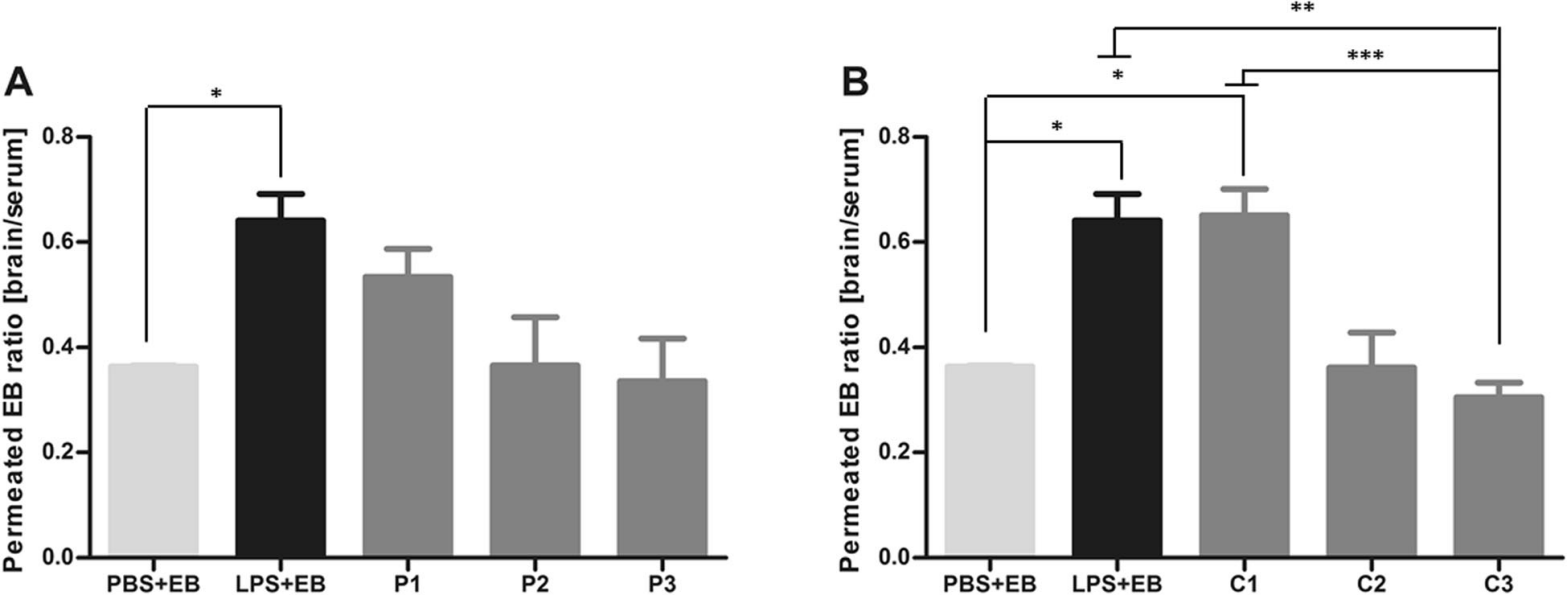
Amount of EB dye penetrated into brain parenchyma. PBS, LPS (1.0 mg/4 ml/kg), and three concentrations of two different kinds of vaccines were injected intraperitoneally. **a** Amount of EB dye after porcine genital respiratory syndrome vaccine (killed-vaccine) injection. **b** Amount of EB dye after canine parvovirus vaccine (live-vaccine) injection. The permeation rate of EB dye in C1 was higher, but not significantly higher than that in LPS/EB group (^*^, *P* < 0.05; ^**^, *P* < 0.01; ^***^, *P* < 0.005)

After i.p injection of PBS, LPS (1.0 mg/4 ml/kg) and two vaccines at three concentrations, the permeability of EB dye in brain parenchyma was measured by the ratio of the amount of EB dye in brain to the amount of EB dye in blood (Fig. 1). In the first injection before EB injection, the result of the group injected PBS was negative control, and the result of the group injected LPS was positive control. When compared to the PBS/EB group, the permeability of EB dye was significantly increased in mice of the group injected with LPS/EB (Figs.1a and b). The EB dye permeability of P1 group (; injected with 2-fold concentration of porcine vaccine stock solution) and P2 group (; injected with reference concentration of porcine vaccine stock solution) was increased than PBS/EB group, but not significantly, and EB dye penetrated through the brain parenchyma less than LPS/EB group (Fig. 1a). However, the permeation rate of EB dye in C1 group (; injected with 2-fold concentration of canine vaccine stock solution) was not significant but higher than LPS/EB group (Fig. 1b). The 10-fold diluted group of the two vaccines had similar or lower EB dye permeability than the PBS/EB group.

### Quantification of ZO-1 and occludin mRNA expressions in the brain

**Fig. 2:**
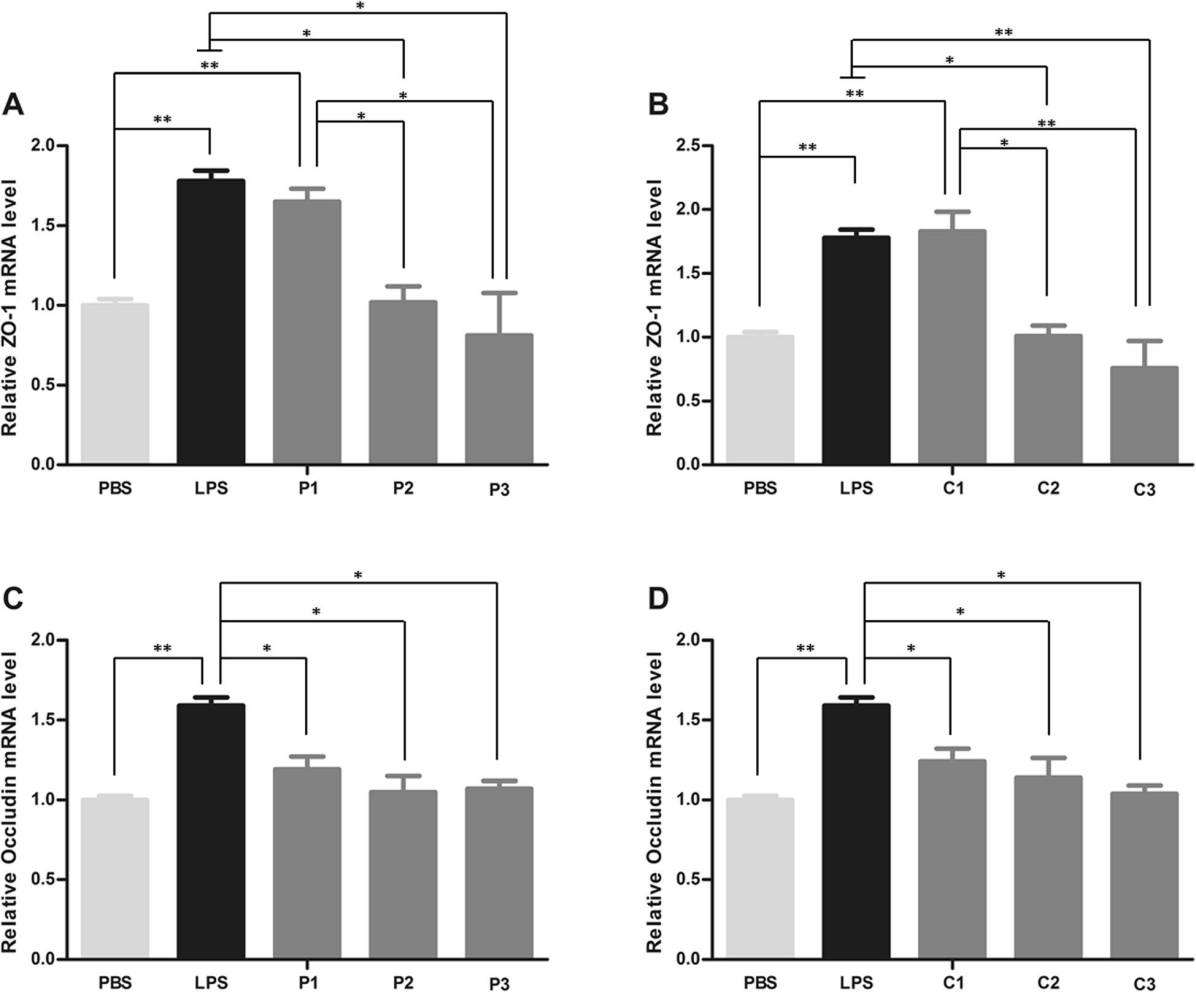
Quantification of mRNA levels of zonula occludens-1 (ZO-1) and occludin in mouse brains. BBB breakdown and restoration were confirmed through expression level analysis of tight junctions in BBB. **a** ZO-1 mRNA expression after porcine genital respiratory syndrome vaccine (killed-vaccine) injection. **b** ZO-1 mRNA expression after canine parvovirus vaccine (live-vaccine) injection. **c** Occludin mRNA expression after porcine genital respiratory syndrome vaccine (killed-vaccine) injection. **d** Occludin mRNA expression after canine parvovirus vaccine (live-vaccine) injection (^*^, *P* < 0.05; ^**^, *P* < 0.01)

The relative ZO-1 and occludin mRNA levels were plotted on the graph after 4 h after i.p injection with PBS, LPS, and two vaccines at three concentrations (Fig. 2). ZO-1 mRNA levels were significantly higher in the LPS group (positive control) than in the PBS group (negative control). In addition, ZO-1 mRNA levels were significantly increased compared to PBS group when 2-fold concentrations of two vaccine stock solutions were injected. At the 2-fold concentration of the stock solution, ZO-1 mRNA expression was lower in the P1 group (; porcine vaccine) but, higher in the C1 group (; canine vaccine) than the LPS group, but not significantly. The ZO-1 mRNA expressions were similar or slightly higher in the P2 and C2 groups (; reference concentration of two vaccines stock solution) compared to the PBS group. And the ZO-1 mRNA expression was similar or slightly lower in the P3 and C3 groups (; 10-fold diluted concentration of two vaccines stock solution) compared to the PBS group.

**Fig. 3:**
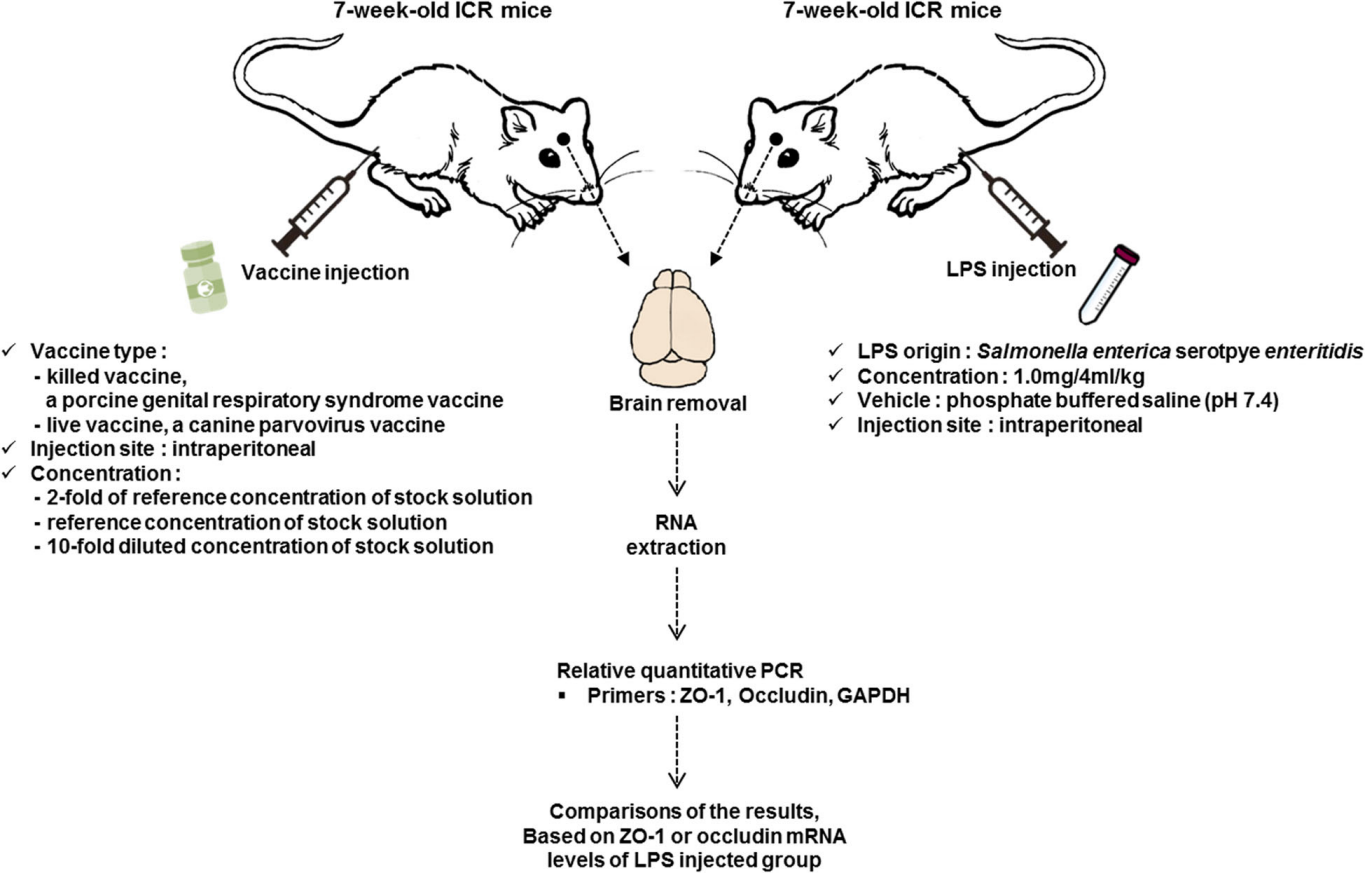
It is a safety evaluation method of vaccine against pre-established brain parenchyma. Based on the results of our established experimental conditions according to *Baek* et al. [1], LPS (S. enterica-derived) and commercially available vaccines are injected intraperitoneally into 7-week-old ICR mice (1.0 mg LPS/4 ml/kg) and brain hemispheres were removed after 4 h post-injection. The mRNA is extracted from the brain, and sequentially performed real time PCR (ZO-1 and occluding). The tight junction mRNA levels of the vaccine-treated group with those of the LPS-treated group were compared and potential risk to the BBB of the vaccine is evaluated. (This conceptual diagram is shown by applying the method used by Baek et al. in [1])

Likewise, occludin mRNA levels were significantly higher in the LPS group (positive control) than in the PBS group (negative control). However, when 2-fold concentrations of two vaccines stock solutions were injected, occludin mRNA expression was increased compared to the PBS group, but not significantly. In addition, the increase was smaller than ZO-1 mRNA level. In the P2, C2, P3 and C3 groups injected with reference concentrations and 10-fold diluted concentrations of the two vaccines, occludin expression was similar or slightly higher than the PBS group.

## Discussion

Humans and live stocks are easily exposed to the risk of infectious diseases caused by various micro-organisms such as bacteria and virus on a daily basis. This has become a very important issue as it affects health in our daily lives [1]. Once a high contagious infectious disease occurs, it can spread to other countries in an instant [4]. Thus, it is not limited to any region or country. Rapid supply of vaccines that are effective in emergency situations is essential to prevent the spread of such infectious diseases [15]. However, in urgent situation, both quick supply of vaccine and safe vaccine are important. All important biological actions of humans and live stocks are regulated and maintained primarily by the central nervous system (CNS), especially the brain [1, 14, 16]. Brain is a complex network of numerous neurons. It is an important organ for regulating body homeostasis, behaviors, memory, and learning [20–22]. This important organ is protected by a structure known as the blood-brain barrier (BBB) [8]. Neurons in the brain and spinal cord are in directly contact with cerebrospinal fluid (CSF) [19]. They are supplied with oxygen and nutrients. When blood and CSF circulate, the material can selectively penetrate because the BBB limits mass transfer into brain parenchyma [11, 18]. It is believed that the brain can examine components of blood and only let required substances selectively pass [2]. If the BBB is exposed to any toxic substance, chronic high blood pressure, radiation, viruses, or bacteria, it can lead to brain problem [3, 18]. If a newly developed vaccine or urgent vaccine fails to secure brain safety, brain parenchyma might become damaged and the health of the recipient may not be assured [1]. This can become can be a serious public health hazard and result in serious economic damage to industrial animals. Therefore, the safety assessment module we designed was focused on how to effectively measure effects of various vaccines on the stability of BBB in laboratory animals [1]. The BBB is formed by a tight junction between endothelial cells of blood vessels distributed throughout the CNS. It contains junctional molecules (JAM), zonula occludens (ZO), claudin, and occluding [3, 5–7, 9–12]. These structures are key components of tight junction of BBB [3, 5–7, 9, 10]. In our previous study, we have established a condition for positive control that can destroy the BBB structure by damage to the tight junction using LPS mentioned earlier [1]. These conditions include mouse species, age, type, concentration of LPS, injection routes to mice, and exposure time after injection. EB assay, qPCR, and Western blot analysis can be conducted under the condition of positive control to determine whether the BBB is destroyed [2, 3, 9, 11, 13, 17, 18]. In the present study, we confirmed that these established conditions and modules of the positive control that destroyed the BBB could be used to assess brain safety of commercial vaccines [1].

Results of EB assay revealed that EB dye permeability of P1 group was higher than that of the PBS/EB group but lower than that of the LPS/EB group. Based on this, we could not conclude that the BBB was damaged in the vaccine concentration of the P1 group. On the other hand, the permeability of EB dye in the C1 group was similar or higher than that in the LPS/EB group, meaning that BBB was damaged at vaccine concentration of C1 group. The same concentration of vaccine was injected. However, higher permeability of EB dye was seen in the C1 group. This result may be due to different vaccine types of porcine and canine vaccines. Since the porcine vaccine is a killed-vaccine and the canine vaccine is a live-vaccine, the canine vaccine is more damaging to BBB. This was also confirmed by qPCR results of ZO-1 mRNA levels. As expected, at reference concentration and 10-fold diluted concentration of stock solution of two vaccines, EB dye permeability and relative ZO-1 and occludin mRNA levels were comparable to those of the negative control, meaning no significant damage to the BBB. Occludin mRNA expression was lower than ZO-1 mRNA expression level at 2-fold diluted concentration of the stock solution which was considered to be damaging to BBB [1]. We have previously examined permeability of EB dye and changes in mRNA and protein expression of ZO-1 and occludin over time after LPS injection. After 4 h of LPS injection, ZO-1 mRNA levels were dynamic and rapidly increased. The level of occludin mRNA increased after 4 h of injection, but increased rapidly after 24 h. These results are highly correlated with our previous findings. However, in order to provide a rapid supply of urgent vaccines, rapid verification is required. Considering this, 4 h after injection of LPS was established as a positive control condition of the module for evaluating BBB damage. Consistent with results of previous studies, ZO-1 levels, but not occludin levels, were increased rapidly after 4 h of vaccine injection. Therefore, when a commercially available vaccine is applied to our positive control and rapid safety assay modules, ZO-1 tight junction primer is more suitable for qPCR. In addition, we have purposed three assays of EB assay, qPCR, and western blot as methods to determine damage to BBB in our previous study [1]. However, since this module is aimed at assessing the safety of urgent vaccines, western blot analysis which requires a relatively long time might not be suitable. Therefore, EB assay and qPCR would be more effective methods for rapid evaluation. It is recommended that we refer to an illustration of our research process on the safety assessment of vaccines (Fig. 3).

## Conclusion

In this study, we confirmed that the safety assessment module for urgent vaccines could be used to evaluate two commercial vaccines. However, in order for this module to be used in practice, it is necessary to apply more types of vaccines to the module. Since there are many kinds of vaccines, they should be repeatedly applied and evaluated using this module. Taken together, the usefulness of the rapid brain safety test module that we constructed previously was verified using vaccines commercially available in the veterinary market in Korea. Our results were highly reproducible. Therefore, this evaluation module can be utilized in the future to assess the safety of various vaccines and adjuvants.

## Data Availability

There was some supporting data available for this work. The datasets used and/or analyzed in this study are available from the corresponding author on reasonable request.
